# The Cut-Off for Triglyceride-Glucose-Body Mass Index (TyG-BMI) and Triglyceride-Glucose-Waist Circumference Index (TyG-WC) Discriminating the Insulin Resistance Based on the SHBG Level and HOMA-IR Value in Caucasian Women with Polycystic Ovary Syndrome

**DOI:** 10.3390/medicina62020291

**Published:** 2026-02-01

**Authors:** Tahar Ben Rhaiem, Aleksander Jerzy Owczarek, Mariusz Wójtowicz, Dariusz Ciura, Paweł Madej, Magdalena Olszanecka-Glinianowicz, Jerzy Tadeusz Chudek

**Affiliations:** 1 Department of Gynecological Endocrinology, Faculty of Medical Sciences in Katowice, Medical University of Silesia in Katowice, 40-055 Katowice, Poland; benrhaiem66@interia.eu (T.B.R.); pmadej@interia.pl (P.M.); 2 Health Promotion and Obesity Management Unit, Department of Pathophysiology, Faculty of Medical Sciences in Katowice, Medical University of Silesia in Katowice, 40-055 Katowice, Poland; aowczarek@sum.edu.pl (A.J.O.); dciura@sum.edu.pl (D.C.); 3 Clinical Department of Gynecology and Obstetrics, Faculty of Medical Sciences in Zabrze, Medical University of Silesia in Katowice, 40-055 Katowice, Poland; mariuszwojtowicz007@onet.eu; 4 Department of Internal Medicine and Oncological Chemotherapy, Faculty of Medical Sciences in Katowice, Medical University of Silesia in Katowice, 40-055 Katowice, Poland

**Keywords:** insulin resistance, TyG-BMI index, TyG-WC index, PCOS, glucose, lipids

## Abstract

*Background and Objectives*: Cut-off points for the triglyceride-glucose-body mass index (TyG-BMI) and the triglyceride-glucose-waist circumference index (TyG-WC) have been established for the assessment of insulin resistance (IR) only in the population of Asian women with polycystic ovary syndrome (PCOS). Therefore, the present study aimed to estimate the cut-off value for these indices discriminating the IR based on the homeostatic model assessment for insulin resistance (HOMA-IR) and sex hormone binding globulin (SHBG) levels established previously in Caucasian women with PCOS. *Material and Methods*: The medical records of 264 selected young adults (18–40 y.o.) Caucasian women with PCOS were the source of parameters: age, body weight, height, waist circumference, glucose, insulin, triglyceride, and SHBG levels, used for calculation of TyG-BMI and TyG-WC indices. The cut-off values for TyG-BMI and TyG-WC indices were calculated using receiver operating characteristic curve analysis. *Results*: The study group included 68 overweight (25.8%) and 62 overweight (23.4%) women. The empirical optimal cut-off values for TyG-BMI and TyG-WC indices corresponding to HOMA-IR values ≥ 2.1 were 233 and 735 [area under the curve (AUC) 85.1% and 86.7%, accuracy 0.814 and 0.784, sensitivity 66.3% and 67.3%, specificity 90.4% and 84.9%, PPV 80.2% and 72.5%, NPV 82.0% and 81.5%], respectively. The empirical optimal cut-off values for TyG-BMI and TyG-WC indices corresponding to SHBG levels < 41.5 nmol/L were 230 and 734 (AUC 79.5% and 77.1%, accuracy 0.735 and 0.723, Se 57.4% and 57.4%, Sp 87.3% and 85.2%, PPV 79.5% and 76.9%, NPV 70.4% and 69.9%), respectively. *Conclusions*: The cut-offs for the TyG-BMI and TyG-WC indices discriminating IR in young Caucasian women with PCOS were similar regardless of whether they are based on HOMA-IR values or SHBG levels.

## 1. Introduction

Polycystic ovary syndrome (PCOS) is an endocrine-metabolic disease that occurs in 5–10% women of reproductive age, and is one of the most frequent causes of sterility [1]. It has been shown that genetic predisposition, obesity, especially visceral, and its complications, including insulin resistance (IR), play a key role in the pathogenesis of PCOS. Insulin resistance and its consequence, hyperinsulinemia, are key pathomechanisms of hormonal (hyperandrogenemia, decreased SHBG and IGF-binding protein levels) and metabolic disturbances (impaired glucose tolerance, type 2 diabetes mellitus, and cardiovascular disease) [2].

The triglyceride-glucose (TyG) index was recently proposed as a surrogate of liver insulin resistance (IR) and steatosis. This index assumes that hepatic IR, resulting in impaired oxidation and utilization of fatty acids and triglycerides, predicts IR. In addition, the process of gluconeogenesis is intensified in subjects with hepatic IR, and the storage of glucose in the form of glycogen in the liver is impaired, which causes an increase in the concentration of fasting serum glucose [3,4].

Based on the TyG index values and the anthropometric parameters body mass index (BMI), waist circumference (WC), and waist–hip ratio (WHR), three novel indices for evaluating IR have been proposed. These indices are calculated by multiplying the TyG index value by the respective anthropometric parameters. An analysis of the 2007–2010 Korean National Health and Nutrition Examination Survey, including 11,149 adults, indicated that the TyG-BMI index is a more optimal, than the TyG index, marker of IR [5]. In turn, in a Chinese cohort of 1544 first-degree relatives of type 2 diabetic subjects, the TyG-WC index was found to be an effective predictor of the risk of the development of diabetes [6]. Moreover, an analysis of the data derived from 1727 adult participants of the National Health and Nutrition Examination Survey (NHANES) conducted in 2017–2018 indicated that TyG-WC, TyG-WHR, and TyG-BMI indices may be used in screening for non-alcoholic fatty liver disease (NAFLD) and metabolic dysfunction-associated fatty liver disease (MAFLD) and monitoring the disease progression. The optimal cut-off points were 822, 4.94, and 238, respectively [7]. Similar cut-off points were shown in a study conducted in Iran in overweight or obese individuals, where the optimal cut-off points for predicting NAFLD and liver fibrosis were 876 for the TyG-WC index (sensitivity 81.3% and specificity 52.3%) and 259 for the TyG-BMI index (sensitivity 78.3% and specificity 51.3%) [8]. Based on the data from 14,498 reproductive-age participants of the 2013–2018 NHANES study, higher TyG-BMI index values were found to be positively correlated with infertility [9].

To date, cut-off points for the TyG-BMI and the TyG-WC indices have been established for the assessment of IR in Asian women with polycystic ovary syndrome (PCOS) [10,11]. In previous studies, a cut-off for the homeostatic model assessment for IR (HOMA-IR) was estimated using sex hormone-binding globulin (SHBG) levels for the diagnosis of liver IR in young Caucasian women with PCOS (>2.1 and <41.5 nmol/L, respectively) [12,13]. To date, these are the only cutoff points for HOMA-IR and SHBG estimated in a population of young Caucasian women with PCOS. Furthermore, they were estimated by our team in previous years, allowing them to be extrapolated to the population of young Polish women with PCOS, who were also our next study group. Therefore, the aim of the present study was to estimate the cut-off values for the TyG-BMI and TyG-WC indices discriminating the IR based on previously established HOMA-IR value and SHBG level in Caucasian women with PCOS.

## 2. Materials and Methods

The present retrospective study includes data from the medical records of 311 consecutive Caucasian women for the first time diagnosed with PCOS according to the Rotterdam criteria [14], who were hospitalized at the Department of Gynecological Endocrinology in 2019–2021.

The inclusion criteria were the diagnosis of PCOS, regardless of phenotype, age 18–40 years, and a complete medical records dataset necessary for this analysis. The exclusion criteria included other endocrinological disturbances (based on routine performed hormonal tests and physical examination), type 1 and 2 diabetes mellitus, dyslipidemia, arterial hypertension, and any pharmacological therapy, non-pharmacological and pharmacological treatment of obesity, and changes in body weight during the last 3 months (based on medical history). The dataset included age, body weight, height, waist circumference, and routine measurements of fasting glucose, triglycerides, insulin, and SHBG levels; all were performed in a single hospital laboratory using the same set of methods for all study subjects. Serum glucose and triglyceride concentrations were measured using the colorimetric method (Roche reagents, Cobas c111; test numbers for glucose 4657527190 and triglycerides 04657594190). SHBG and insulin levels were determined using the ECLIA method (Roche Diagnostics GmbH, Mannheim, Germany, reagents for Cobas e411; test numbers for SHBG 750 and insulin 650) [15].

This retrospective analysis was approved by the Bioethical Committee of Silesia in Katowice (CBN/0022/KB1/97/21 date 7 April 2021).

### 2.1. Analysis Flow

The reasons for excluding data from the analysis were described in our previously published study [12]. The reasons for excluding 47 women from the study were the following: thyroid diseases (N = 35), type 1 and 2 diabetes (N = 7), and arterial hypertension (N = 8), as well as the use of pharmacotherapy for them. Finally, data from 264 (84.9%) women were analyzed. The cut-off point for IR was HOMA-IR > 2.1 [12]. The second marker of IR was SHBG concentration < 41.5 nmol/L [13].

### 2.2. Data Analysis

BMI, HOMA-IR, and TyG index values were calculated with the following standard formulas:

HOMA-IR = fasting insulin level (µIU/mL) × fasting glucose level (mg/dL)/405

TyG-BMI = Ln [fasting triglycerides level (mg/dL) × fasting glucose level (mg/dL)/2] × BMI

TyG-WC = Ln [fasting triglycerides level (mg/dL) × fasting glucose level (mg/dL)/2] × WC

Impaired fasting glucose was diagnosed based on the classification of diabetes mellitus from 1997, fasting serum glucose level from 100 mg/dL to 125 mg/dL [16].

### 2.3. Statistical Analysis

Statistical analysis was performed using STATISTICA 13.0 PL (TIBCO Software Inc., San Ramon, CA, USA), StataSE 13.0 (StataCorp LP, College Station, TX, USA), and R software (R version 4.5.1 (2025-06-13 ucrt)—“Great Square Root”, R Core Team 2013; R: A language and environment for statistical computing. R Foundation for Statistical Computing, Vienna, Austria. URL http://www.R-project.org/ (accessed on 28 January 2026)). Statistical significance was set at a *p*-value < 0.05. All tests were two-tailed. Imputations were not performed for missing data. Nominal and ordinal data were expressed as percentages. Interval data were expressed as median with lower and upper quartiles. The distribution of the variables was evaluated by the W Shapiro–Wilk test and the quantile-quantile (Q-Q) plot. Data were presented as mean ± standard deviation in case of normal data distribution or as median (lower quartile–upper quartile) for data with non-normal or skewed distribution. To identify a cut-off point discriminating the IR based on the HOMA-IR value and SHBG level, parametric and non-parametric receiver operating characteristic (ROC) curves were calculated with the area under the curve (AUC) and corresponding sensitivity, specificity, positive and negative predictive value, as well as with the accuracy of classification. These parameters were calculated based on the true positive (TP), true negative (TN), false positive (FP), and false negative (FN) numbers of subjects classified according to HOMA-IR and SHBG levels. Accuracy was calculated as (TP + TN)/N. To identify an optimal, empirical cut-off point value for the TyG-BMI and TyG-WC indices, the Youden J statistic (index) was used.

## 3. Results

Finally, after considering the inclusion and exclusion criteria, data from 264 women from 311 available records were analyzed. The study group characteristics, including PCOS phenotypes, are summarized in Table 1. More than 50% of the subjects exhibited visceral obesity and impaired fasting glucose, while overweight and obesity, according to BMI, were noted in ~25% of patients. Hypertriglyceridemia was noted in 9.5% of patients, IR, assessed by HOMA-IR, in 37.1%, and IR assessed by SHBG levels in 46.2% of study women.

**Figure 1:**
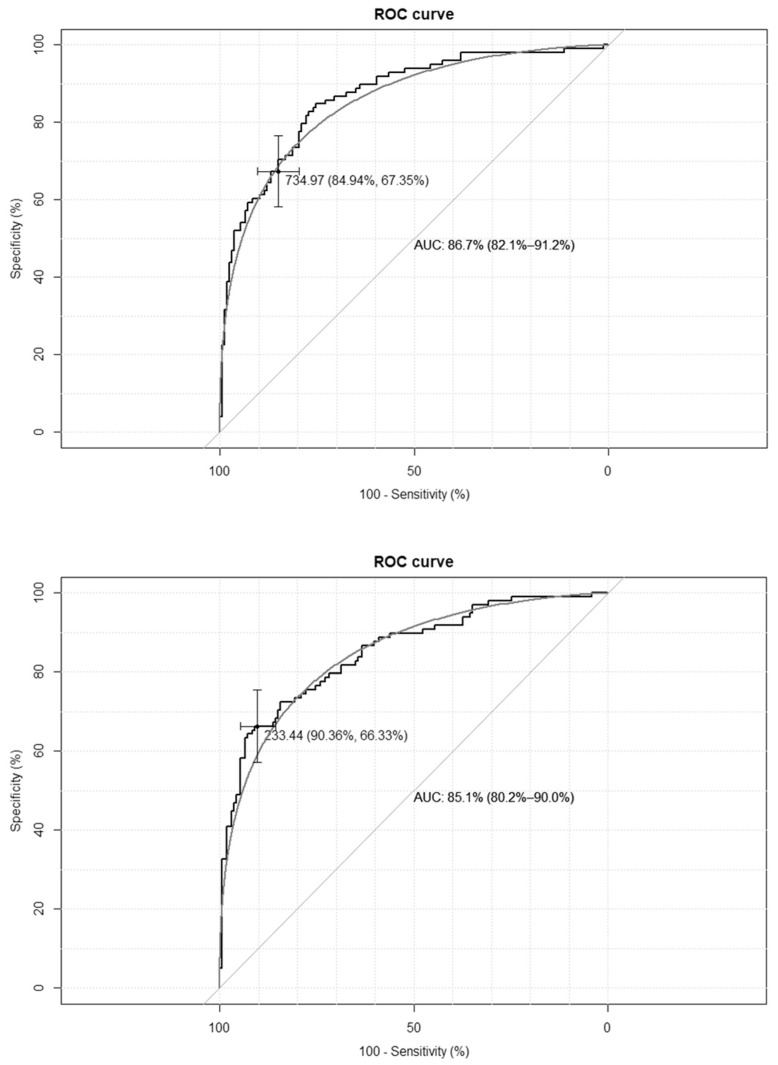
Receiver–operating characteristic curves (ROC) for the cut-off point of the triglyceride-glucose-body mass index (TyG-BMI) index value for the diagnosis of insulin resistance based on homeostasis model assessment of insulin resistance (HOMA-IR) value > 2.1 (**upper panel**) and based on sex hormone binding globulin (SHBG) concentration < 41.5 nmol/L (**lower panel**) in the group of 264 women with polycystic ovary syndrome. AUC—area under the curve. The black line depicts the empirical ROC curve, while the gray line based on the binomial distribution.

**Figure 2:**
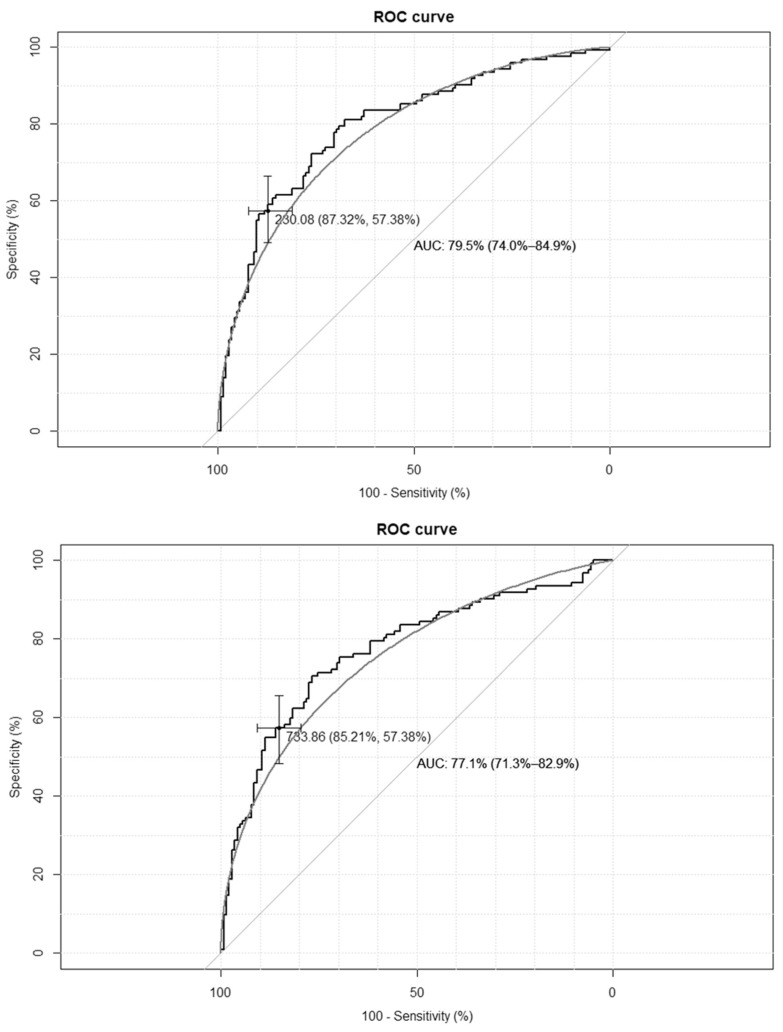
Receiver–operating characteristic curves for the cut-off point of triglyceride-glucose-waist circumference index (TyG-WC) value for the diagnosis of insulin resistance based on homeostasis model assessment of insulin resistance (HOMA-IR) value > 2.1 (**upper panel**), and based on sex hormone binding globulin (SHBG) concentration < 41.5 nmol/L (**lower panel**) in the group of 264 women with polycystic ovary syndrome. AUC—area under the curve. The black line depicts the empirical ROC curve, while the gray line based on the binomial distribution.

The empirical optimal cut-off values for the TyG-BMI index, corresponding to HOMA-IR values ≥ 2.1 and SHBG levels < 41.5 nmol/L, were 233 and 230, respectively, while for the TyG-WC index, corresponding to HOMA-IR values ≥ 2.1, and for the SHBG levels < 41.5 nmol/L, these were 735 and 733, respectively. The ROC curves are presented in Figure 1 and Figure 2.

The clinical credibility, based on the calculated cut-off points, is presented in Table 2. The TyG-BMI index achieves the optimal accuracy, with the highest specificity and both the best positive and negative predictive values.

There was a very high positive correlation between the TyG-BMI index and the TyG-WC index (r = 0.92; *p* < 0.001). By calculating the sum of the TyG-BMI and TyG-WC indices, there were 160 (60.6%) subjects with negative results for both indices, corresponding to an HOMA-IR and SHBG insulin-resistant state, 24 (9.1%) with one of the indices positive, and 73 (27.7%) with both indices as positive. This indicates a strict concordance between the classification of IR based on the HOMA-IR and SHBG cut-off levels, following the sum of TyG-BMI and TyG-WC indices over the established cut-off values in ROC analysis (Kappa = 0.970, 95% confidence interval: 0.948–0.992).

## 4. Discussion

To the best of our knowledge, our study is the first to estimate the cut-off values for the TyG-WC index used to discriminate IR in women with PCOS worldwide. Moreover, for the first time, we estimated the cut-off values for the TyG-BMI index in Caucasian women with PCOS. To date, these points for the TyG-WC index were estimated in the general population [7,8,9]; the cut-off values for the TyG-BMI index have been investigated solely in Asian women with PCOS [10,11].

The previously empirically estimated cut-off points in Caucasian women with PCOS were 2.1 for HOMA-IR [12] and 41.5 nmol/L for the SHBG levels [13]. Based on these cut-off points, the present study demonstrated that the estimated cut-off points for TyG-BMI and TyG-WC indices were calculated. These cut-off points for the TyG-BMI index were similar to those estimated in the general USA population for early screening of NAFLD and MAFLD (233 and 230 vs. 238), while for the TyG-WC index, these were significantly lower (735 and 733 vs. 822) [7]. The cut-off points for the TyG-WC index were also slightly lower than those estimated in a Chinese cohort of 1544 first-degree relatives of patients with type 2 diabetes for the assessment of the development of type 2 diabetes (735 and 734 vs. 760) [6]. These differences may be due to both racial variability and the way the TyG-WC index cut-off point was empirically estimated. In the present study, the references were markers of IR, including the HOMA-IR value and SHBG level. In contrast to these observations, the USA study utilized elastography, and the Chinese study examined the risk of developing diabetes [6,7]. Moreover, our study included only young women, whereas previous studies involved both women and men, as well as young and older subjects [6,7]. The sensitivity of both cut-off points for the TyG-WC index estimated in this study was lower than that reported in the Chinese study (67.3% and 57.4% vs. 74.7%), yet a higher specificity (84.9% and 85.2% vs. 63.1%) was observed [6]. It should be noted that both cut-off points for the TyG-BMI index were higher than those estimated in Chinese women with PCOS (233 and 230 vs. 191), while sensitivity was lower (66.3% and 57.4% vs. 85.3%) and specificity was higher (90.4% and 87.3% vs. 73.9%) [10]. Of interest, both cut-off points for the TyG-BMI index were similar to the estimated in Bangladeshi women with PCOS (233 and 230 vs. 235), while sensitivity was higher for the cut-off point estimated based on HOMA-IR values (66.3% vs. 60%) and lower for the cut-off point estimated based on SHBG levels (57.4% vs. 60%), and specificity was much higher for both cut-off points estimated in our study (90.4% and 87.3% vs. 74%). In turn, both cut-off points for the TyG-WC index estimated in our study were slightly higher than in Bangladeshi women with PCOS (735 and 734 vs. 720), while sensitivity was lower (67.3% and 57.4% vs. 74%) and specificity was higher (84.9% and 85.2% vs. 63%) [11].

It should be noted that in our study, the cut-off point values for both TyG-BMI and TyG-WC indices differed slightly between the two methods of estimation based on HOMA-IR and SHBG cut-off points for IR, yielding a very high concordance between the methods examined. Based on sensitivity, specificity, AUC value, and accuracy, it was recommended to use the cut-off point for the TyG-BMI index of 233 and for the TyG-WC index, the cut-off point of 735 in young Caucasian women with PCOS. The use of these indices together with the TyG index as a verification of screening test may reduce the costs of diagnosing IR based on HOMA-IR and SHBG levels. The use of three indices in screening will reduce the risk of false-negative results. The results from the NHANES study also suggest that in women with PCOS with TyG-WC index values above the cut-off point, liver imaging tests should be performed to assess their steatosis and fibrosis [7].

In addition to our previously determined cut-off point for TyG [15], the estimated cut-off points can be used as a screening test for IR in the Polish Caucasian women with PCOS. Furthermore, abnormal values may indicate the need for body composition testing and the detection of fatty liver disease, as well as therapeutic interventions to reduce body fat mass. It should also be noted that recently published studies performed in the cohort of Chinese women with PCOS showed that elevated TyG-BMI values have a negative impact on ovulation [17], adverse pregnancy outcomes [18], and in vitro fertilization outcomes [19], as well as the rate of live birth in fresh embryo transfer cycles [20].

The relatively large size of the study group, including Polish Caucasian women with PCOS, is the strength of the present study. Polish women are representative of the typical for Caucasian non-Hispanic European race; thus, the cut-off points established in the present study may be extrapolated at least for Central and Eastern Europeans of the Caucasian race.

The main limitations of our study include the retrospective design and the lack of a control group. Future studies should be conducted prospectively, include follow-up, and a control group. Several years of observation would be necessary to see how these cut-off points change depending on fluctuations in body weight in both women with and without PCOS. Another limitation is the lack of diagnosis of MAFLD and liver fibrosis, and the use of biochemical tests performed as part of routine clinical diagnostics at different time points for the analyses. In addition, the effect of diet and physical activity on glucose and triglyceride levels was not analyzed. It should be noted that specific dietary errors, such as increased consumption of sweets, fruit, honey, or alcohol, as well as decreased or increased physical activity on the day or a few days preceding the test, can affect glucose and triglyceride levels and, consequently, TyG index values. This can contribute to both false-positive and false-negative diagnoses.

As mentioned, there are no estimated TyG-BMI and TyG-WC cut-off points for the general Caucasian population or for individuals with pathogenic disorders involving IR, such as prediabetes or hypertension. The cut-off points provided in our study may serve as a starting point for further research in these populations.

## 5. Conclusions

The cut-offs for the TyG-BMI and TyG-WC indices discriminating insulin resistance in young Caucasian women with PCOS are similar regardless of whether they are based on HOMA-IR values or SHBG levels.

## Figures and Tables

**Table 1:** Characteristics of the study group of PCOS women [N = 264].

Parameters	
Age [years]	26 + 5
Phenotypes [N/(%)]	
A	155 (58.6)
B	31 (11.8)
C	40 (15.3)
D	38 (14.3)
Body weight [kg]	68.0 (59.0–83.9)
BMI [kg/m^2^]	24.8 (21.3–29.4)
Overweight (BMI 25–29.9 kg/m^2^) [N/(%)]	68 (25.8)
Obesity (BMI ≥ 30 kg/m^2^) [N/(%)]	62 (23.4)
Waist circumference [cm]	84.9 ± 15.9
Visceral obesity (WC > 80 cm) [N/(%)]	144 (54.6)
Triglycerides [mg/dL] (NR: <150 mg/dL)	95.3 ± 49.0
Hypertriglyceridemia (≥150 mg/dL) [N/(%)]	25 (9.5)
Glucose [mg/dL] (NR: 70–99 mg/dL)	84.9 ± 5.8
Impaired fasting glucose (100–125 mg/dL) [N/(%)]	157 (59.5)
Insulin [µIU/mL] (NR: 2–10 µIU/mL)	7.9 (5.6–12.0)
HOMA-IR	2.2 ± 2.0
HOMA-IR ≥ 2.1 [N/(%)]	98 (37.1)
SHBG [nmol/L] (NR: 34.3–147.7 mmol/L)	46.4 (30.2–64.8)
SHBG ≤ 41.5 nmol/L [N/(%)]	122 (46.2)
TyG-BMI index	2045 (171–248)
TyG-WC index	667 (576–787)

Mean ± standard deviation for data with normal distribution or median [lower quartile–upper quartile] for data with non-normal or skewed distribution. Abbreviations: BMI—body mass index; HOMA-IR—homeostasis model assessment of insulin resistance, NR—normal range; SHBG—sex hormone binding globulin; TyG-BMI—triglyceride-glucose-body mass index; TyG-WC—triglyceride-glucose-waist circumference index.

**Table 2:** Results of the receiver operator curves analysis and clinical credibility based on the calculated cut-off points for homeostasis model assessment of insulin resistance (HOMA-IR) value ≥ 2.1 and sex hormone binding globulin (SHBG) concentration < 41.5 nmol/L.

	HOMA-IR ≥ 2.1		SHBG < 41.5 nmol/L	
	TyG-BMI	TyG-WC	TyG-BMI	TyG-WC
Area under the curve	85.1%	86.7%	79.5%	77.1%
	(80.2–90.0%)	(82.1–91.2)	(74.0–84.9%)	(71.3–82.9%)
Cut-off value	≥233	≥735	≥230	≥734
Accuracy	0.814	0.784	0.735	0.723
Sensitivity	66.3%	67.3%	57.4%	57.4%
	(56.0–75.4%)	(57.0–76.3%)	(48.1–66.2%)	(48.1–66.2%)
Specificity	90.4%	84.9%	87.3%	85.2%
	(84.6–94.2%)	(78.4–89.8%)	(80.4–92.1%)	(78.1–90.4%)
Positive predictive value	80.2%	72.5%	79.5%	76.9%
	(69.6–87.9%)	(62.0–81.1)	(69.3–87.1%)	(66.7–84.8%)
Negative predictive value	82.0%	81.5%	70.4%	69.9%
	(75.4–87.1%)	(74.7–86.8)	(63.0–76.9%)	(62.4–76.5%)
Diagnostic odds ratio	18.46	11.63	9.27	7.46
	(9.50–35.88)	(6.39–21.18)	(5.03–17.08)	(4.32–13.94)
*p* value	<0.001	<0.001	<0.001	<0.001

## Data Availability

The raw data supporting the conclusions of this article will be made available by the authors on request.

## Abbreviations

The following abbreviations are used in this manuscript:

AUC	Area under the curve
BMI	Body mass index
HOMA-IR	Homeostasis model assessment of insulin resistance
MAFLD	Metabolically-dysfunctional-associated fatty liver disease
NAFLD	Non-alcoholic fatty liver disease
NHANES	National Health and Nutrition Examination Survey
NPV	Negative predictive value
PCOS	Polycystic ovary syndrome
PPV	Positive predictive value
ROC	Receiver-operating characteristics
SHBG	Sex hormone binding globulin
TyG	Triglyceride-glucose index
TyG-BMI	Triglyceride-glucose-BMI index
TyG-WC	Triglyceride-glucose-WC index
WC	Waist circumference
WHR	Waist-hip ratio
